# Put your feet up: The impact of personality traits, job pressure, and social support on the need for recovery after work

**DOI:** 10.1007/s12144-022-02950-1

**Published:** 2022-03-14

**Authors:** Knut Inge Fostervold, Reidulf G. Watten

**Affiliations:** 1 Department of Psychology, University of Oslo, P.O. Box 1094, Blindern, N-0317 Oslo, Norway; 2 Department of Psychology, Inland Norway University of Applied Sciences, P.O. Box 400, Elverum, Norway

**Keywords:** Need for recovery, Five-factor model, Job pressure, Social support, Work-related fatigue

## Abstract

The need for recovery after work (NFR) is an important warning of work-related fatigue. NFR is linked to prolonged work-related efforts and depletion of resources, creating a need for temporary respite from work demands. The aim of the current study was to investigate the relationships between NFR and the five-factor model (FFM), comprising the personality traits of emotional stability (ES), extraversion (E), agreeableness (A), conscientiousness (C), and openness to experience (O). Perceived job pressure and perceived social support were included as mediators. The study was conducted using structural equation modelling (SEM) on cross-sectional data from a sample of 681 participants from several work sectors (N females = 376, N males = 305; *M* age = 46.9 years; *SD* = 11.1). The results showed that NFR was affected both directly and indirectly by FFM traits. High ES and high O contributed directly to reduced and increased NFR, respectively. High perceived social support contributed to reduced NFR, while high perceived job pressure contributed to increased NFR. High ES contributed indirectly to reduced NFR through perceived job pressure and social support, high O contributed indirectly to increased NFR through perceived social support, and high E contributed indirectly to increased NFR through perceived job pressure. A and C were not related to NFR. The findings demonstrate that personality traits, especially ES, are firmly related to NFR and highlight the importance of incorporating personality factors into studies of work environmental factors on NFR.

**Supplementary Information:**

The online version contains supplementary material available at 10.1007/s12144-022-02950-1.

## Introduction

Work inevitably entails demands that require personal effort, which can induce adaptive psychological and bodily reactions (e.g., changes in motivation and attention, as well as elevated adrenaline, corticosteroids, and blood pressure). Prolonged effort leads to the depletion of psychological resources associated with the short-term and long-term costs of coping with work demands, creating a desire for a temporary respite from work, denoted as the ‘need for recovery after work’ (NFR) (Meijman & Mulder, 1998; van Veldhoven, 2008). According to the effort-recovery model (Meijman & Mulder, 1998), the main driver of NFR is not the demand itself, but rather the effort each individual invests in meeting a specific demand. The depletion process is thus a reaction related to coping with loads of actual work demands, the mental load connected to the use of compensatory strategies, and load remnants from previously unsuccessful attempts to recover (Wentz et al., 2020). NFR is accordingly expected to fluctuate across time and situations. NFR is not necessarily a problem in itself, as the depletion process is reversible. However, if the day-to-day workload continues without the necessary time and opportunity for adaptive reactions to be recuperated, NFR will accumulate over time. Endured over longer periods, this may increase personal effort even more and escalate into permanent work-related fatigue.

Work-related fatigue seems to be quite widespread in the general population. A European investigation comprising more than 21,000 employees showed that over 40% felt their daily amount of work was too high (Paoli & Merllié, 2001). Additionally, studies on professional sectors, such as medicine and health care, show elevated levels of work-related fatigue (Brzozowski et al., 2021; Ho et al., 2013; Smith-Miller et al., 2014). Work-related fatigue is associated with a number of health issues such as psychosomatic complaints, psychological distress, reduced health, and reduced well-being (Bültmann et al., 2002; Rydstedt et al., 1998; Sluiter et al., 2003), and consequently represents a public health issue, with considerable impact on national health care systems and societies in general.

Although, NFR and work related fatigue are acknowledged as different constructs (Jansen et al., 2002), NFR is an early indicator of work-related fatigue. The association between these two factors seems firmly established in the research literature. Sonnentag and Zijlstra (2006) for example, found a strong association (β = .69) between work-related fatigue and NFR. Identifying psychological antecedents and correlates of NFR is therefore important not only to enhance our understanding of why NFR differs among workplaces, work tasks, and individuals but also for reasons of prevention.

Both prolonged efforts and the depletion process contain elements of self-regulation that could be influenced by personality traits. For instance, recent research has shown that neuroticism is associated with poor self-regulation in general and especially with respect to decision making and emotional coping strategies (de la Fuente et al., 2020). However, personality may also influence NFR indirectly through other work-related variables. Job pressure and social support are recognized key variables in the stress process and, therefore, prone to affect the effort – recovery relationship and the level of NFR (Härmä, 2006). Individual perceptions of both variables are important in the appraisal of work demands and consequently crucial for the amount of coping effort invested by the employee. Thus, perceived job pressure and perceived social support are liable to the influence of personality factors (Berg et al., 2005; Zellars & Perrewé, 2001). Hence, it is reasonable to assume that personality traits could influence NFR through perceived job pressure and perceived social support.

## Theoretical Model

The effort-recovery model emphasizes work-related factors, such as decision latitude and job pressure, to explain the amount of NFR experienced by an individual. Although psychological dispositions have been deemed important in the model (Meijman & Mulder, 1998), the influence of personality factors seems to have been largely overlooked (van Veldhoven, 2008). It is well known that personality influences work-related perceptions, attitudes, cognitions, and behaviour (e.g., Törnroos et al., 2013). It is thus reasonable to expect that personality also influences the experience of NFR, the quality of the recovery process, and the amount of restitution needed. Further, every organisation is unique in its composition of persons, work tasks, and the environment. Coinciding patterns exist in how people perceive, interpret and react to events, situations and conditions, and such patterns are influenced by personality (Serfass & Sherman, 2013). Hence, if the goal is to enhance our understanding of how work affects health and well-being, the effect of personality traits should be included in the analyses.

### Personality Traits and NFR

The FFM is the most recognized structural description of personality used today. In the FFM, personality is conceived of as a combination of five dimensions: extraversion (E), agreeableness (A), conscientiousness (C), emotional stability (ES) (the opposite pole of neuroticism), and openness to experience (O) (McCrae & Costa Jr, 1997). Personality traits are rather stable across time and situations (McCrae & Costa Jr, 1997), but will always interact with other situational, organisational, and sociocultural factors.

The literature on possible links between FFM traits and NFR is sparse. To our knowledge, only one study thus far has examined the relationship between FFM traits and NFR. In that study, ES, E, and C were included in a regression model, predicting impaired work functioning in a sample of depressed employees in remission. Although univariate regression effects were present, the results showed that none of the FFM traits were retained in the final prediction model (de Vries et al., 2015). However, the sample size was rather low (*N* = 68) and encompassed a clinical sub-group (major depressive disorder [MDD]) that might not reflect a general working-life population. Other studies have shown associations between FFM traits and work-related factors linked to NFR, such as burnout (Armon et al., 2012; Bakker et al., 2006), fatigue (Calderwood & Ackerman, 2011; De Vries & Van Heck, 2002), work-related stress (Grant & Langan-Fox, 2007; Vollrath, 2001), and psychological detachment (Naseer et al., 2012). To propose a theoretical model, it is thus necessary to draw inferences from studies addressing the relationship between FFM traits and constructs related to NFR and work environmental factors in general. In the following, we delineate more detailed, hypothesized links between FFM traits and NFR*.*

#### Hypothesized Associations between FFM Traits and NFR

E is generally associated with favourable outcomes such as positive affect, high job satisfaction, and a general feeling of well-being (Grant & Langan-Fox, 2007; Maggiori et al., 2016). In reviewing the literature, Jackson and Schneider (2014) conclude that high E seems to alleviate feelings of stress by problem-focused coping and stressor appraisals, emphasizing the positive aspects of a stressful situation. High E has previously been associated with reduced fatigue (De Vries & Van Heck, 2002; Poeschla et al., 2013). High E is also associated with high effort at work and high reward from work (Törnroos et al., 2012). Providing that the mentioned factors also affect perceptions of NFR, high E should predict reduced NFR.

ES may be of special interest as this trait encompasses emotional adaptation and stability versus maladjustment and lability. In cognitive stress tasks, individuals with low ES (high neuroticism) apply dysfunctional coping strategies, such as avoidance and emotion-focused coping, instead of the more adequate task-oriented coping shown by individuals with high ES (low neuroticism) (Boyes & French, 2010). These findings are also supported by neuro-biological evidence describing negative attention bias and interpretation of information and increased reactivity (Ormel et al., 2013). Moreover, elevated neuroticism is associated with higher frequency of daily hassles and perceived exhaustion (Schmidt et al., 2017) as well as high work effort at work, but low reward (Törnroos et al., 2012). It is therefore not surprising that the research consistently finds low ES to be associated with low job satisfaction (Bruk-Lee et al., 2009). Thus, due to its association with dysfunctional coping strategies (Boyes & French, 2010), negative affectivity (Tackett & Lahey, 2017), physiological and psychological stress reactions, threat appraisal, poor task performance, and autonomic pathophysiological reactivity (Schneider, 2004; Schneider et al., 2012), it is reasonable to expect that low ES leads to increased NFR due to greater work-related efforts and more depletion of resources.

O reflects aesthetics and intellectual stimulation, tolerance, autonomy, and interest in the unknown. Individuals with high O are characterized by acceptance, curiosity, creativity, tolerance for new ideas, introspection, openness to sensations, and cultural differences (Connelly et al., 2014; McCrae & Costa Jr, 1997). Research findings show associations between high O and increased experiences of fatigue (De Vries & Van Heck, 2002), and reduced psychological detachment from work (Naseer et al., 2012), considered a core element in the recovery process. Low levels of detachment from work have been associated with increased job stress and workload, and have predicted high strain and poor well-being (Sonnentag & Fritz, 2015). Although some findings are mixed and to some extent contradictory (Hildenbrand et al., 2018; Lü et al., 2016), it is not unreasonable to expect that elevated O is related to increased NFR.

For the remaining two factors, A and C, the literature is limited. Both traits are considered important in modern working life. People high in A are cooperative, empathic, and friendly, while those high in C are characterized by self-discipline and motivation through targeted activities, orderliness, and reliability (McCrae & Costa Jr, 1997). Low A has been associated with chronic fatigue syndrome (Nater et al., 2010), and high C can predict reduced fatigue (Calderwood & Ackerman, 2011; De Vries & Van Heck, 2002; Sørengaard et al., 2019). Moreover, both A and C have been associated with reduced use of emotional coping (Fornés-Vives et al., 2016) and increased psychological detachment from work (Naseer et al., 2012). Thus, it seems likely that high A and high C could imply lowered NFR.

As summarized, our main hypothesis is:

- *H1: Extraversion, emotional stability, agreeableness, and conscientiousness will be negatively associated with NFR, and openness will be positively associated with NFR.*

### NFR, Perceived Job Pressure, and Perceived Social Support

Job pressure is the result of demands linked to the job itself, such as deadlines, working hours, frequent interruptions, and the number of work tasks. In the effort-recovery model (Meijman & Mulder, 1998), perceived job pressure can be viewed as a cognitive appraisal of job demands. Job pressure is consequently recognized as a risk factor for NFR (Härmä, 2006; Sluiter et al., 2003).

Social support denotes the perception or reality that one is cared for and can rely on assistance from others. In work settings, the term usually includes support from colleagues and supervisors. In the effort-recovery model (Meijman & Mulder, 1998), perceived social support can be viewed as a job resource, mitigating the negative impact of high job demands. Social support has been associated with a range of positive outcomes, which also apply to NFR (Gommans et al., 2015; Sluiter et al., 2001; Wentz et al., 2020). High perceived social support should accordingly be associated with reduced NFR. Our hypothesis in relation to job pressure and social support is therefore as follows:

- *H2: Perceived job pressure will be positively related to NFR, and perceived social support will be negatively related to NFR.*

#### Indirect Effects of Personality on NFR

Personality is likely to influence both perceived job pressure and perceived social support and thereby NFR. For instance, in the literature, high ES has been associated with reduced perceived job pressure due to factors such as less emotional exhaustion (Liu & Yu, 2019), more functional coping strategies and better self-efficacy beliefs in how to deal with negative emotions (Alessandri et al., 2018). Social support is also influenced by ES. Low ES (high neuroticism) seems to reduce perceived social support, while elevated ES has the opposite effect, probably linked to the differences in affect display (Swickert et al., 2010). Individuals with low ES will frequently exhibit reactions such as irritability, ruminations, easily upset, etc., and can create a negative emotional atmosphere and stressful working environment (Törnroos et al., 2013), contrary to high ES which is associated with calmness and stability, thereby creating other social relations. Thus, ES is probably positively related to social support.

Persons with high O, showing rich fantasy life, awareness of emotions, liberal values, curiosity, etc., perceive their work as less demanding and stressful (Törnroos et al., 2013). It could be related to their cognitive flexibility and imaginative intellect making them more resilient to work stress, but at the same time vulnerable for engagement in too many activities and too much work (van Emmerik, 2008). However, their creativity, originality, and unconventional style could have negative consequences for social support. The behaviours and expressions exhibited by those high in O may be considered to be too unfamiliar and provoking by many people and may reduce their willingness to provide social support for high O people (Swickert et al., 2010). The O-trait might thus be negatively associated with social support.

For the remaining traits (E, A, and C), the research findings are somewhat equivocal. E is generally linked to positive affect, gregariousness, and positive co-worker relations (Bowling et al., 2005). However, E is also associated with assertiveness, activity, and sensation seeking. This may motivate extraverts to take on too many work tasks, especially when colleagues and peers reinforce those choices. The research has shown that this may in fact be the case. Swickert et al. (2002) found that the E-trait was positively correlated with stress but that this relationship was mediated through perceived availability of social support, especially belonging support. Others have found high E to be associated with increased perceived workload (Chiorri et al., 2015) and increased perceived challenging job demands (Rudolph et al., 2017). Although some studies has reported no, or only trivial, associations between E and job pressure (Törnroos et al., 2013; van Emmerik, 2008), high E may be associated with increased job pressure due to increased job engagement and devoted energy among extraverts (Young et al., 2018).

With regard to A, the literature is scarce. Törnroos et al. (2013) found that high A was associated with reduced job strain and lower effort-reward imbalance. Anand et al. (2015) showed that employees’ ability to cope with job related stress was dependent upon their level of A. High A moderated the mediated family-work-stress relationship resulting in better coping, a buffering effect. Thus, the A-trait could be indirectly associated with NFR through job pressure and social support.

Persons with elevated C-scores are competent, industrious, disciplined, reliable and motivated through targeted activity (McCrae & Costa Jr, 1997), and they show good academic and job performance (Conard, 2006; Witt et al., 2002). However, the relationship between C and job pressure has been scantly studied. In some studies, C has been linked to reduced perceived workload and job strain as would be expected from core elements in the trait such as competence (Chiorri et al., 2015; Törnroos et al., 2013; Zellars et al., 2006), but also due to their good social functioning and effective coping strategies (Barańczuk, 2019). These characteristics might reduce their need for social support, although they might perceive the availability for support. Corresponding negative associations are consequently conceivable for C on job pressure and social support.

Summarized, the hypotheses within the personality trait domain affecting job pressure and social support variables are:

- *H3: Emotional stability, conscientiousness, and agreeableness will be indirectly related to NFR through a negative association with perceived job pressure, and openness and extraversion will affect NFR indirectly through a positive association with job pressure.*
- *H4: Emotional stability, extraversion, agreeableness, and conscientiousness will be indirectly related to NFR through a positive association with perceived social support, and openness will affect NFR indirectly through a negative association with social support.*

## Materials and Methods

### Design and Procedures

Three reasons justify the cross-sectional design used in the current study:

- Firstly, adult personality traits are understood as stable dispositions in cognition, emotion and behaviour, which are congenital or formed early in life (McCrae & Costa Jr, 1997; Mõttus et al., 2017). NFR, however, is considered a transient phenomenon that varies on a daily basis (Meijman & Mulder, 1998). This also applies to the relationships between personality traits and variables measuring perceived job demands. The possibility of reversed causality (i.e. that NFR could change personality traits) is therefore quite unlikely since the transient nature of NFR cannot be compared to the long-term impact of major life events that can influence personality, and which most likely occur in young or old ages (Specht et al., 2011). The causal direction between job demands and NFR is similarly presupposed by the effort-recovery model (Meijman & Mulder, 1998).
- Secondly, as mentioned above, the relationship between personality and NFR has, to date, not been thoroughly investigated, and the pattern of covariation among these sets of variables therefore remains unknown. Establishing relations between variables is therefore the first step in building more complex models and at this stage a cross-sectional design is useful (Spector, 2019).
- Thirdly, we still have no knowledge of a potential timeframe during which the relationships among the variables develop. In this case we study behavioural and emotional states that might vary in levels over time, but the temporal precedence of the factors is difficult to assess. Instead of exploring all potential models, the overarching aim in structural equation modelling is to test plausible theoretical models empirically. By utilizing the cross sectional design it is thus possible to rule out alternative explanations and test possible explanations according to our hypotheses.

The data were collected using an electronically distributed questionnaire. A total of 28 office organisations agreed to participate in the study. The management of each organisation distributed an email containing written information about the study to all employees. The email stated the purpose of the study, that participation was voluntary, and that answering the questionnaire was allowed during normal working hours. A follow-up email included a link to the electronic questionnaire, which was located on a secure website. Completed questionnaires were saved anonymously. A reminder was sent by email to all employees two weeks after the first invitation.

### Participants

A total of 1919 invitations were distributed; 681 participants completed the questionnaire (35.1%; N females = 376; N males = 305). The sample included participants employed in organisations from both the public (76.2%) and private sectors (23.8%) located in the southeastern part of Norway. The work tasks were predominately ordinary office work and encompassed public administration, accounting, research and education, consultancy, finance, real estate, and public and private agencies. The mean age was 46.9 years (SD = 11.1), the mean tenure was 12.8 years (Mdn = 8; SD =12.9), and 80.3% of the sample had an education beyond the high school level. Cell offices were the most common workspaces (69%), while the remainder (31%) worked in open plan offices.

### Measures

The first part of the questionnaire contained background variables (age, gender, tenure, education, and office type). The rest of the questionnaire consisted of Norwegian versions of internationally validated scales.

#### Personality Traits – Five-Factor Model

The FFM traits were measured by the Norwegian version of the Big-Five Inventory (BFI-44) (Engvik & Føllesdal, 2005). The BFI consists of 44 statements designed to measure five personality traits: ES (neuroticism reversed), E, A, C, and O. Following the manual, the respondents were asked to consider each statement on a Likert scale ranging from 1 = *disagree strongly* to 7 = *agree strongly*. Sample questions are: E) “Is talkative” and “Generates a lot of enthusiasm”, ES) “Is relaxed, handles stress well” and “Remains calm in tense situations”, A) “Is helpful and unselfish with others” and “Has a forgiving nature”, C) “Does a thorough job” and “Is a reliable worker”, and O) “Is original, comes up with new ideas” and “Has an active imagination”.

Internationally, the BFI has shown satisfactory reliability, with coefficient alpha values (α) between .75 and .90, and a test-retest reliability (*r*) between .80 and .90. Reliability estimates for the Norwegian edition are comparable, although somewhat lower (Engvik & Føllesdal, 2005). The coefficient alpha values (α) obtained in the present sample (estimates reported by Engvik and Føllesdal (2005) are set forth in parentheses): E: .83 (.82), A: .72 (.75), C: .79 (.81), ES: .84 (.84), and O: .82 (.81).

#### Job Pressure and Social Support

Subjectively perceived job pressure and social support were measured by two subscales from the Norwegian edition of the Job Stress Survey (JSS-N) (Spielberger & Håseth, 2004). Each subscale was measured twice, with 10 items representing important stressors or lack of support in the work environment. Severity was measured on a nine-point scale from 1 = *low stress* to 9 = *high stress*. The frequency of workplace stressors or lack of support in the last 6 months was measured on a ten-point scale from *0 days* to *9+ days*. The job pressure index and the social support index were calculated as the product of the intensity and frequency of the items. In accordance with the JSS manual, social support was measured as a lack of support. The lack of support index was reversed before conducting the analyses in order to agree with the format usually present in the literature. Examples of items measuring job pressure were “Assignment of increased responsibility” and “Making critical on-the-spot decisions”. Examples of items measuring lack of support were “Inadequate support by supervisor” and “Lack of recognition for good work”. In the present sample, the coefficient alpha (α) for the job pressure index was .83, and .86 for the lack of support index, which is comparable to the normed data (.81 and .86, respectively) (Spielberger & Håseth, 2004).

#### Need for Recovery after Work

The need for recovery after work (NFR) was measured by a Norwegian version of the Need for Recovery Scale (van Veldhoven & Broersen, 2003). The scale was translated and back-translated from English into Norwegian following the principles of psychometric equivalence. The scale consisted of 11 items measuring daily fatigue and recuperation from work with a categorical yes/no response option. The number of yes responses were summed to form a single index. Sample items were “I find it difficult to relax at the end of a working day” and “When I got home, people should really leave me alone for some time”. The coefficient alpha in the present sample was .89, which aligns with the value (.88) reported in a large Dutch sample (van Veldhoven & Broersen, 2003), and is somewhat higher than the alpha value (.83) obtained in a heterogeneous sample of white-collar workers from the UK (Devereux et al., 2011). Results by de Croon et al. (2006) showed stable test-retest values for NFR in situations where the work environment is considered to be stable, while at the same time being sensitive and able to show changes in workload over time.

### Common Method Bias

Common method bias (or variance) (CMB) is suspected to inflate the correlations between constructs, especially in studies utilizing self-report as the sole data collection method (Podsakoff et al., 2012). Identification of the criterion (dependent) variable and its expected behaviour are considered key elements in the formation of both CMB and participant reactivity in general (Podsakoff et al., 2012; Spector, 2006). All participants in the present study were informed that the study’s main purpose was to investigate how personality affects different factors in the work environment. Based on this information and the magnitude and variety of the constructs measured, it is unlikely that the participants were able to single out NFR as the criterion variable. It is also unlikely that the participants, based on their answers to previous scales, would be able to generate systematic hypotheses about the desired answers to subsequent scales. Further, the main predictors and the criterion variable were spatially separated and used different response scales (Likert versus dichotomic yes/no). Both are procedural measures assumed to remedy the danger of CMB (Conway & Lance, 2010; Podsakoff et al., 2012). The possibility of a pervasive CMB factor was further examined by a Harman’s Single Factor Test (Fuller et al., 2016). The principal axis factor (PAF), including all items assessing the predictor- and criterion variables, explained 12.7% of the variance. Thus, even though an acknowledged threshold for pervasive CMB is lacking, the result does not indicate that CMB constitutes a substantial problem in the current measurements.

### Statistical Analyses

IBM SPSS version 26 was used for descriptive analyses and the bivariate intercorrelations (*r*) among the study variables. Structural equation modelling (SEM), with maximum likelihood parameter estimation, was used to examine the empirical relationship between the predictor variables and NFR. The analyses were conducted in IBM AMOS version 26.

Confirmatory factor analyses (CFA) were conducted for all scales included in the model (See supplementary material). The results showed that especially the FFM dimensions and perceived social support achieved unsatisfactory fit. Inspecting the items representing each construct shows pairs of items with highly correlated error terms, indicating that the measured constructs are complex and multidimensional. However, the multidimensional nature of the FFM is well known in the literature (e.g., Vassend & Skrondal, 1997; Wright, 2017). This is also the case in most scales measuring perceived social support (e.g., Osman et al., 2014) and the Job Stress Survey is no exception in this regard (Holmström et al., 2008; Spielberger & Håseth, 2004). All the included scales have been previously validated and commonly used and there was no intention to examine or modify the properties of the measurement models representing each scale in the current study. Poorly fitted measurement models are prone to influence the fit of the structural model (Williams et al., 2009). Thus, to maintain psychometric rigor and simultaneously include measurement error in the analyses of predictors, mediators, and outcome variables, the measurement constructs were modelled as single-indicator latent factors. The advantages and disadvantages of this approach, as well as its use, have been discussed by scholars such as Hayduk and Littvay (2012) and Savalei (2019).

The index score, calculated according to the scoring manual for each measure, was used as an observed single indicator. The error variance for each single indicator (Var(∊_i_)) was set according to the formula [($1-{\widehat{\mathrm\rho}}_{{\mathrm y}_{\mathrm i}{\mathrm y}_{\mathrm i}}$var(y_i_)] (Bollen, 1989). For the sake of consistency, and because the present reliability estimates were highly comparable with reliability estimates that have been obtained in previous research, the reliability estimates obtained in the present study were used when calculating the error variance.

The goodness-of-fit of the structural model was evaluated using chi-square (χ^2^), the comparative fit index (CFI), the Tucker-Lewis index (TLI), the root mean square error of approximation (RMSEA; with high and low values of the 90% confidence interval [CI]), and the standardized root mean square residual (SRMR). The cut-off criteria for good model fit were a CFI and TLI above .95, an RMSEA below .05, and an SRMR value below .08 (Hu & Bentler, 1999). The Bayesian information criterion (BIC) was used for model comparison. Lower BIC values imply improved fit. According to Raftery (1995), a difference in BIC values between 6 and 10 is considered a strong indication of a meaningful difference between the models, and a difference larger than 10 is considered very strong.

Bootstrap estimation, using bias-corrected 95% confidence intervals, was applied to test the significance of indirect effects. The estimates were based on 5000 bootstrap samples generated by random sampling with replacement from the data (Cheung & Lau, 2008).

#### Potential Moderating Effects of Control Variables

Moderator relationships may exist irrespective of main effects and may influence the stability of structural models and should be considered in the analysis. With regard to personality and NFR, gender, work sector (private or public), and workspace (cell office or open plan office) may be of particular interest. Gender and work sector are both recognized as potential moderator variables in work settings (Armon et al., 2012; Liu et al., 2008; Markovits et al., 2010). Workspace is known to affect work environmental variables such as stress, fatigue, and social relationships (James et al., 2021). All the three variables seem to imply personality differences (Grönlund & Magnusson, 2018; Maczulskij, 2017; Seddigh et al., 2016).

The possibility of a moderating effect of gender, work sector, and workspace was examined by fitting three multigroup models to the final model. In the first model, the parameters were allowed to vary freely across gender. The structural path coefficients were then constrained to be equal, and the difference in goodness-of-fit between the unconstrained and constrained models was calculated and evaluated following the recommendations of Chen (2007). Corresponding analyses were conducted across work sectors (private or public) and workspaces (cell office or open plan office).

#### Potential Careless Responders

To ensure the quality of the data, the sample was examined for careless responders by scrutinizing suspicious outliers and participants with unusually brief response times. Furthermore, we calculated the standard deviation for each participant and inspected the results for participants showing zero variance in at least one of the included scales. A total of 73 participants were identified as potentially careless responders. However, distinguishing between true careless responders and participants with viable responses is difficult. There are responders who actually have zero variance on some scales, not because they are careless, but because that is how they perceive and respond to the questions. Following the recommendation from Niessen et al. (2016), the dataset was cleaned of all potential careless responders and the results compared to the results from the entire dataset. The results are reported in the results section.

## Results

### Descriptive Analyses

The results from the correlational analysis indicated that the demographic variables of gender (*r* = −.03), work sector (*r* = .03), age (*r* = −.06), tenure (*r* = .01), and education (*r* = −.03) were only weakly associated with NFR. After testing for potential suppressor effects, we excluded demographic variables from the main analysis. Table 1 portrays the means, standard deviations, and bivariate correlations (*r*) between all variables included in the study.

**Table 1:** Personality traits, perceived social support, perceived job pressure and need for recovery. Means, standard deviations and zero-order correlations (*r*) are among the study variables

Variable	*M*	*SD*	1.	2.	3.	4.	5.	6.	7.	8.	9.	10.	11.	12.	13.	14.
1. Gender			–													
2. Work Sec.			−.06	–												
3. Worksp.			.06	−.51**	–											
4. Educ.			.15**	−.11**	.13**	–										
5. Age	46.9	11.1	.01	.15**	−.23**	−.36**	–									
6. Tenure	12.8	12.9	−.01	.18**	−.23**	−.51**	.67**	–								
7. E	4.49	1.06	−.07	−.16**	.11**	.15**	−.08*	−.10*	–							
8. A	5.47	0.75	−.14**	.10*	−.01	−.14**	.06	.04	.20^**^	–						
9. C	5.35	0.83	−.18**	.15**	−.05	−.09*	.11**	.08*	.11^**^	.40^**^	–					
10. ES	5.02	1.07	−.11**	−.03	.06	.01	.12**	.04	.29^**^	.45^**^	.40^**^	–				
11. O	4.89	0.97	.15**	−.09*	.04	.34**	−.11**	−.21**	.35^**^	.07	−.01	.11^**^	–			
12. JP	18.66	11.47	.03	−.09*	.24**	.15**	−.21**	.20**	.04	−.10^**^	−.11^**^	−.19^**^	.07	–		
13. SS	67.60	11.89	.05	.10**	−.06	−.16**	.11**	.10**	−.02	.14^**^	.08*	.19^**^	−.10^**^	−.42^**^	–	
14. NFR	3.14	3.00	−.03	.03	.06	−.03	−.06	.01	−.14^**^	−.22^**^	−.18^**^	−.41^**^	.08^*^	.31^**^	−.31^**^	–

Included variables: *Work Sec.* work sector (private/public), *Worksp.* workspace (cell/open plan office), *Educ.* education, *E* extraversion, *A* agreeableness, *C* conscientiousness, *ES* emotional stability, *O* openness to experience, *JP* perceived job pressure, *SS* perceived social support, *NFR* need for recovery after work. *p < .05, **p < .01

As shown in Table 1, the bivariate correlations between the predictor variables and NFR were significant. Significant intercorrelations were also present between the dimensions of FFM, except A by O and C by O. The strongest relationship (*r* = .45) was found between the traits of ES and A.

### Proposed Structural Equation Model

Based on the theoretical model and our research hypotheses, a model with NFR as the criterion variable was drawn. The five dimensions of the FFM were considered to be exogenous predictor variables in the model. Perceived job pressure and perceived social support were entered as endogenous predictor variables. Hypothesized direct causal paths were drawn between each predictor variable and NFR.

Research has long shown that the FFM dimensions are intercorrelated (McCrae et al., 2008). Thus, bidirectional paths were added to the model between A, C, and ES and between E and O, representing the inter-correlations often identified as the two higher-order factors of personality (Digman, 1997). Bidirectional paths were also drawn between E and ES, C, and A to account for the pattern of intercorrelations commonly reported among these dimensions of the FFM (Grant & Langan-Fox, 2007; Törnroos et al., 2013; Van der Linden et al., 2010; Vittersø, 2001). Finally, a bidirectional path was added between perceived job pressure and perceived social support to account for the close relationship repeatedly reported between the two variables (Haly, 2009; Karasek & Theorell, 1992).

Examination of the theoretical model’s goodness-of-fit measures revealed a significant chi-square (χ^2^(3) = 11.00, *p* = .012), which implies that the hypothesis of exact fit was rejected. However, this result should be expected given the sample size of the present study. Further, the results showed that the CFI (.99) was above the threshold of .95, while the TLI (.91) was below this threshold. The RMSEA (.063, 90% CI [.026, .104]) was above the criterion of good fit. The SRMR (.025) was below the criterion of .08. The BIC value was 226.274.

### Final Structural Equation Model, Constraining Non-significant Paths

Inspection of the parameter estimates of the model revealed that several of the hypothesized paths were not significant. To develop a more parsimonious model, the non-significant paths were constrained to zero one at a time, and the parameters were re-estimated for each step. New paths or correlations were not fitted to the model during this process. Thus, all modified models were nested within the proposed structural equation model.

After revision, the influence of A and C were no longer retained in the model. The final model’s goodness-of-fit measures indicated a non-significant chi-square χ^2^(11) = 17.61, *p* = .091). The other fit measures showed a close fit with the empirical data (CFI = .99, TLI = .98, RMSEA = .030, 90% CI [.000, .054], SRMR = .026). The BIC value was 180.700.

**Fig. 1:**
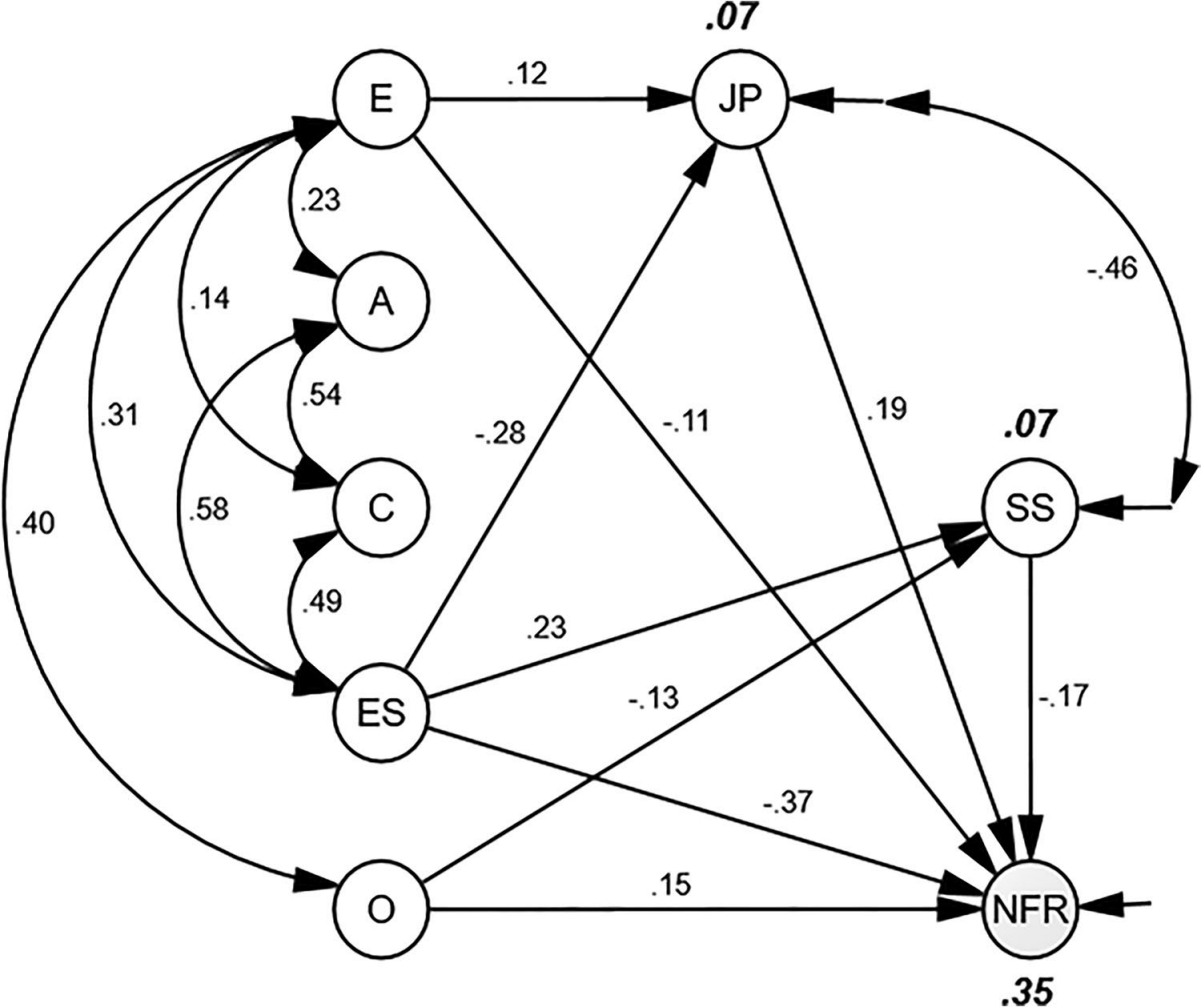
Final model depicting structural relationships among exogenous predictor variables, endogenous predictor variables, and need for recovery after work. Exogenous predictor variables: E = extraversion; A = agreeableness; C = conscientiousness; ES = emotional stability; O = openness to experience. Endogenous predictor variables: JP = perceived job pressure; SS = perceived social support. Criterion variable: NFR = need for recovery after work. Numbers on arrows are standardized regression coefficients. Large, bold, italicized numbers are R^2^ (explained variance) in latent variables (JP: 7%, SS: 7%, NFR: 35%). Paths constrained to zero, empirical indicators, and error terms are omitted to enhance readability

Comparing the statistics for the theoretical model and the final model demonstrated minor changes for the CFI and the SRMR, while the RMSEA (ΔRMSEA = .033) and the TLI (ΔTLI = .07) were substantially improved. The chi-square difference test was not significant (Δχ^2^ = 6.61, Δdf = 3, *p* = .085), which suggests a similar fit for the two models. In such cases, the most parsimonious model is usually preferred (i.e., the model comprising the fewest estimated parameters). The reduced BIC value found for the final model (BIC_diff_ = 45.574) substantiates this interpretation, and denotes very strong support for a meaningful difference between the two models (Raftery, 1995). Therefore, the final model, explaining 35% of the variance in NFR, was retained. Figure 1 presents the final model.

According to Fig. 1, high E was associated with reduced NFR (β = −.11; SE = .049; *p* = .038) and increased job pressure (β = .12; SE = .046; *p* = .010). High ES was associated with reduced NFR (β = −.37; SE = .044; *p* < .001), reduced job pressure (β = −.28; SE = .048; *p* < .001) and increased social support (β = .23; SE = .042; *p* < .001). High O was associated with increased NFR (β = .15; SE = .047; *p* = .002) and reduced social support (β = −.13; SE = .042; *p* = .002). High job pressure was associated with increased NFR (β = .19; SE = .055; *p* = .002), while high social support was associated with reduced NFR (β = −.17; SE = .052; *p* = .001).

#### Indirect Effects

Three FFM traits, E, ES, and O, were indirectly associated with NFR. Table 2 portrays the bootstrapped standardized regression coefficients (β), unstandardized regression coefficients (b), standard errors (SE), 95% CIs, and probability values (p).

**Table 2:** Personality traits and need for recovery. Total and indirect effects are mediated through perceived social support and perceived job pressure. Standardized regression coefficients (β), unstandardized regression coefficients (*b*), standard errors (SE), their associated 95% CIs, and probability values (*p*)

Path	β	*b*	SE	95% CI low-high	*p*
				Indirect effect	
E → JP → NFR	.023	.067	.030	[.018, .140]	.006
ES → JP → NFR	−.053	−.146	.049	[−.257, −.063]	.001
ES → SS → NFR	−.039	−.112	.039	[−.204, −.046]	.001
O → SS → NFR	.022	.070	. 032	[.020, .147]	.002
				Total effect	
E → NFR	−.083	−.243	.143	[−.517, .047]	.094
ES → NFR	−.464	−1.314	.125	[−1.560, −1.073]	< .001
O → NFR	.170	.542	.150	[.251, .840]	< .001

Included variables: *E* extraversion, *A* agreeableness, *C* conscientiousness, *ES* emotional stability, *O* openness to experience, *JP* perceived job pressure, *SS* perceived social support, *NFR* need for recovery after work

As seen in Table 2, all indirect effects retained in the final model were significant (p < .05). High E seems to predict increased NFR indirectly through job pressure (β = .02). ES was mediated by two paths: one through job pressure (β = −.05) and one via social support (β = −.04). In both cases, high ES was associated with reduced NFR. High O was indirectly associated with increased NFR through social support (β = .02).

#### Total Effects

The total effects of the three FFM traits E, ES, and O on NFR are displayed in Table 2. The most prominent effect was found for ES (β *=* −.46), yielding reduced NFR. For trait O, the effect seems to be the opposite (β *=* .17). Although the effect is not as pronounced, high O seems to be associated with increased NFR. The impact of trait E is somewhat more subtle. The direct association between E and NFR revealed a negative relationship (β *=* −.11). At the same time, E was indirectly, positively associated with NFR through perceived job pressure (β *=* .023). As the positive and negative values of the β-coefficients cancel each other out when calculating the total effect, the total effect of E appears small (β *=* −.08) and not significant.

### Stability of the Final Model across Gender, Work Sectors, and Workspaces

Analyses were based on the final model depicted in Fig. 1. The chi-square of the unconstrained cross-gender model was not significant (χ^2^(23) = 25.447, *p* = .328). All other fit measures indicated a good fit with the data (CFI = .99, TLI = .99, RMSEA = .013, 90% CI [.000, .035], SRMR = .030). The difference between the unconstrained model and the model constraining the structural path coefficients to be equal revealed a non-significant chi-square (Δχ^2^ = 14.834, Δdf = 8, *p* = .062). Changes in the other fit measures were small and did not indicate a substantially worse fit (ΔCFI = .007, ΔTLI = .012 ΔRMSEA = -.008, ΔSRMR = - .007).

The chi-square for the unconstrained model across the work sector was not significant (χ^2^(23) = 31.954, *p* = .101). The other indices indicated a good fit (CFI = .99, TLI = .97, RMSEA = .024, 90% CI [.000, .042], SRMR = .047). The difference between the unconstrained model and the model constraining structural path coefficients to be equal was not significant (Δχ^2^ = 6.921, Δdf = 8, *p* = .545). All other measures denoted minor differences in fit (ΔCFI = −.002, ΔTLI = −.009 ΔRMSEA = .005, ΔSRMR = −.011).

Multigroup analyses across workspaces revealed a non-significant chi-square for the unconstrained model (χ^2^(23) = 25.293, *p* = .335). The other indices indicated a good fit (CFI = .99, TLI = .99, RMSEA = .012, 90% CI [.000, .035], SRMR = .026). The difference between the unconstrained model and the model constraining structural path coefficients to be equal was not significant (Δχ^2^ = 7.258, Δdf = 8, *p* = .509). All other measures showed minor differences in fit (ΔCFI = −.001, ΔTLI = −.004 ΔRMSEA = .003, ΔSRMR = −.003).

Thus, the final model in Fig. 1 was stable across gender, work sector, and workspace, demonstrating that neither of the three variables moderated the observed relationship pattern.

### The Impact of Potential Careless Responders

Compared to the entire sample, analyses of bivariate correlations in the sample, cleaned for potential careless responders, showed minor changes, predominately between .01 and .02. The largest change was found for the correlation between perceived job pressure and perceived social support, which declined from *r* = −.42 to *r* = −.38.

The SEM analysis of the sample, cleaned for potential careless responders, revealed a non-significant chi-square χ^2^(11) = 12.649 *p* = .317). The other goodness of fit measures indicated good fit with the empirical data (CFI = .99, TLI = .99, RMSEA = .016, 90% CI [.000, .047], SRMR = .024). Compared to the entire sample, the fit of the cleaned sample was slightly improved. The structural relationships were, for the most part, marginally weaker in the cleaned sample than in the entire sample, both with regard to path coefficients (−.03 at most) and covariances (−.04 at most). Explained variance was reduced with 1%. Thus, cleaning the sample for potentially careless responders did not alter the results substantially.

## Discussion

The aim of the present study was to investigate the influence of personality traits assessed according to the FFM, perceived job pressure, and perceived social support on NFR. The final path model showed satisfactory fit with the empirical data and was stable across gender and work sector.

As summarized, our main hypothesis (H1) was partly confirmed. ES (β = −.37) and E (β = −.11) were directly and negatively associated with NFR, while O (β = .15) was directly and positively associated with NFR. Our second hypothesis (H2) was confirmed: Perceived job pressure (β = .19) was positively associated, and perceived social support (β = −.17) was negatively associated, with NFR. The third hypothesis (H3) was partly confirmed: E was indirectly and positively related to NFR through perceived job pressure (β = .023). ES was indirectly and negatively related to NFR through perceived job pressure (β = −.053). Finally, the fourth hypothesis (H4) was partly confirmed: O was indirectly and positively associated with NFR through perceived social support (β = .022), while the indirect association between ES and NFR, through perceived job pressure, was negative (β = −.039). A and C were unrelated to NFR, both directly and indirectly.

Together with the influence of perceived job pressure and perceived social support, personality traits explained 35% of the variance in NFR. Although this outcome is far from trivial, it implies that most of the variance in NFR is due to other factors not included in the study. Obvious alternatives are a range of known risk factors in the work environment, as laid out by scholars such as Aronsson et al. (2014) and Wentz et al. (2020). The current results underscore that subjective perceptions and appraisals of work environmental factors, such as perceived role stress (Rai & Kumar, 2012) and the level of trust at the workplace (Rahman et al., 2016), should also be accounted for when investigating NFR and work related fatigue.

### The Five-Factor Model and NFR

The obtained results contrast with the findings of de Vries et al. (2015), who only reported univariate relationships between the FFM dimensions of neuroticism (low ES), E, and C in relation to NFR. In our study, personality traits, and most notably ES, were firmly related to NFR both directly and indirectly.

Presumably, individuals with low ES scores are easily ‘triggered’ in periods of stress and high workload, and will therefore experience long-lasting emotional, cognitive, autonomic, and bodily reactions. This view is in line with neuro-psychological evidence showing that elevated neuroticism (low ES) was associated with reduced sustained activation in the orbitofrontal cortex, increased response to emotional arousal in the right medial prefrontal cortex, and attenuated valence processing in the right temporal lobe when viewing emotional pictures (i.e. the brain areas involved in emotional regulation) (Kehoe et al., 2012). Thus, individuals with low ES will tend to respond more strongly to emotionally arousing situations and experiences, and will probably require more time to return to pre-arousal states (i.e., increased need for recovery).

The core elements of low ES may also explain the direct negative relationship with perceived job pressure. This condition leaves individuals prone to increased arousal, which entails low tolerance for stressful situations such as job pressure (van Emmerik, 2008). In addition, individuals who are low in ES more often seem to generate conflicts and emotional discomfort in the work environment (Aeron & Pathak, 2017). These factors could explain the association between ES and perceived social support. The negative emotional and communicative style of individuals with low ES and the associated negative cognitive schemas probably lead them to be vigilant of the possible costs of receiving social support (Park et al., 2013). Evoked reactions from the environment could further exacerbate trait-related stress reactions and increase emotional rumination, leading to greater NFR later on (Hamesch et al., 2014). Hence, people experiencing low ES seem to struggle with emotional activation, which makes them vulnerable to a range of work-related factors. Further research should examine how this affects NFR and their ability to recuperate.

O is probably the least investigated FFM trait, making the present study an important supplement to the existing literature. The results showed that O was positively related to NFR, both directly and indirectly. As such, the results corroborate previous findings indicating negative work-related consequences associated with high O (De Vries & Van Heck, 2002; Naseer et al., 2012).

One explanation could be the motivational aspect of this trait and how people high in O process information. High O seems to strengthen the association between work engagement and performance, at least among students (Bakker et al., 2015). Elevated O also seems to be related to increased tolerance for negative working environmental factors such as noise (Franklin et al., 2013). In addition, tendencies toward cognitive involvement and the pursuit of difficult goals mean that those high in O are less likely to seek out emotional and social support (Righetti et al., 2014) in their work environment. In sum, elevated O-scores could cause individuals to be more inclined to stay at work for longer periods, which increases the risk of work-related fatigue and eventual burnout. This may also explain why high O seems to be associated with reduced social support, and is thereby indirectly linked to increased NFR. Swickert et al. (2010) also suggest that the social unconventionality characterizing O itself may explain the tendency toward reduced social support. The behaviours and expressions exhibited by those high in O may be considered too unfamiliar and provoking by many people, and may reduce their willingness to provide social support to people with high O. Thus, despite having received limited interest in the literature previously, O seems to be an important factor in work settings and deserves further exploration.

E affected NFR negatively via a direct association, and positively by an indirect path through perceived job pressure, thus causing the total effect to be rather low and not significant. The direct association confirms previous studies highlighting positive aspects of the trait, such as positive affect and reduced stress (Jackson & Schneider, 2014). However, the indirect association seems to confirm the reversed side of this trait by corroborating the literature, indicating higher perceived workload among individuals characterized by high E (Chiorri et al., 2015; Rudolph et al., 2017; Swickert et al., 2002). These findings are also supported by the results of Leikas and Ilmarinen (2017), who showed a delayed fatigue reaction among extraverts, and a meta-study by Young et al. (2018), who reported a high degree of job involvement among extraverts. Thus, increased job involvement and engagement could lead to more work, a higher workload, and increased feelings of job pressure, which could thereby indirectly result in greater NFR. Thus, balancing the conflicting propensities of the E trait constitutes a challenge, not only for extraverted people, but also for their colleagues. This matter deserves deeper investigation.

### Job Pressure, Social Support, and NFR

The relationship among perceived job pressure, perceived social support, and NFR corroborates a wealth of research originating from the influential job strain model by Karasek and Theorell (1992). The basic assumption is that unhealthy work environments evolve in situations characterized by high job pressure and little control over how to perform work tasks. Support from others or from the organisation is supposed to affect the relationship between job pressure and control. The current findings are also partly in line with results from a prospective investigation of computer workers (Kraaijeveld et al., 2014). Adverse psychosocial work characteristics, such as high job demands, low job control, and low social support have been found to be related to increased NFR, especially in the case of older workers. The current findings also underscore the importance of incorporating appraisals and individual perceptions of work environmental factors into the effort-recovery model. Work demands can often be described quite objectively. Different individuals may nevertheless perceive the same job demand quite differently, and sometimes even view similar job demands in dissimilar ways on different occasions. To expand upon the effort-recovery model, actual or objective work demands should be recognized as related to, but separate from, perceptions and appraisals of the same phenomenon.

### Theoretical and Practical Implications

According to the effort-recovery model (Meijman & Mulder, 1998), the main driver of NFR is the effort employees invest to cope with current work demands. Wentz et al. (2020) extended the model by including the net effect of effort connected to the use of coping/compensatory strategies and remnants from previous unsuccessful recovery, balanced against the availability of job resources and recovery options during the workday.

The current study expands upon the scope of the model even further by showing that personality affects NFR both directly and indirectly. Personality does not change work demands, but shapes how people think and act relative to work conditions, coping efforts, and off-work activities. Thus, to understand how NFR and work-related fatigue develop and are preserved, personality and other individual factors such as locus of control and coping styles should be taken into account.

However, modern work-life is not only about providing income for subsistence, without taxing consequences for fatigue, health and well-being. Work is also an important arena for forming social identity, social cohesion, feelings of meaning, and the affirmation of personal core values. All individuals deserve the privilege of a healthy work life that is perceived as rewarding and safe until retirement. To be sustainable, working conditions must be adapted to the needs of the individual employee (Fostervold et al., 2018).

The current research highlights how individual differences in personality traits make some employees more vulnerable than others to work demands and the effort needed to cope with such demands. These differences should be attended to by leaders and HR management to prevent negative health consequences, reduced productivity, and early labour marked exclusion. Special care should be given to employees showing signs of low ES, as the emotional reactivity associated with this trait makes them especially vulnerable to ambiguousness in the work environment (Enns et al., 2005). Clear role expectations, job resources matching job demands, and recognition of the concerns they often convey when confronted with changes and new developments will contribute to a more predictable and psychologically safer work environment that may lower NFR.

Employees high in E and O should also be given more attention. Both traits are generally considered advantageous by leaders and colleagues in the workplace. However, as shown in the current study, both traits are associated with increased NFR, although for elevated E the association is indirect, through increased perceived job pressure.

Leaders should nevertheless be attentive to tendencies toward extensive work hours, restrict the possibility to take on extra work, and ensure sufficient time to recover. How this can be achieved in practice will to a large part depend on national regulatory frameworks and organisational policies and culture. A possible tool may be to explore thoughts and feelings around this topic in one-to-one dialogues. For this to be successful, an organisational culture characterized by trust and psychological safety is essential, together with an already demonstrated respectful and genuine interest by the manager into the needs and well-being of the employee.

That said, we would not recommend implementing individual measures based solely on FFM scores. Although presumably valid at the group level, the present knowledge regarding the relation between personality and NFR is still limited at the individual level. Personality traits primarily contain information about general disposition in how people encounter their work environment. It is possible, as shown for other life outcomes (Stewart et al., 2021), that narrower facet- or item-specific information may provide better predictive validity than the five personality domains. Moreover, personality traits are not, and should not, be seen as deterministic typologies. It is known that the situational strength affects trait activation. Strong situations contain information or cues that communicate clear expectations about acceptable behaviours. The resulting psychological pressure tend to restrain the activation of individual dispositional behaviour. The absence of such contextual cues, known as week situations, have an opposite effect that promotes the display of trait specific behaviours (Judge & Zapata, 2015). Thus, future studies should continue to explore how and under what circumstances personality affects NFR at both the group and the individual level.

### Strengths and Limitations

Investigating some traits without controlling for the remaining dimensions may lead to spurious results and leave gaps in our knowledge about how personality relates to NFR (Grant & Langan-Fox, 2007; Lee-Baggley et al., 2005). Hence, we included all five traits in the statistical analyses.

The current study was based on a rather heterogeneous sample, which may increase the generalizability of the findings by reducing the limitations associated with samples from a restricted range of organisational settings. Furthermore, the structural model was stable across gender and work sector and did not change substantially when re-analysed in a sample cleaned for potentially careless responders. Finally, the use of SEM should be considered advantageous compared to the regression analyses often used in previous research. Taken together, this indicates that the final model is robust, and that the observed relationships could also be expected in other populations of office workers.

However, the educational level of the current sample was relatively high, as most participants had education beyond high school. As such, the current findings may be confined to higher educated workers. However, the bivariate relationship between education level and NFR was low and not significant in the current study, a result corresponding to the outcomes reported on highly educated female workers in the Netherlands (Verdonk et al., 2010).

Common method bias (or variance) (CMB) should also be regarded as a potential threat to internal validity in the present study, as in most other research. Although the nature, magnitude, and pervasiveness of CMB is under debate (Conway & Lance, 2010; Spector & Brannick, 2009), several measures were implemented during the data collection process to counteract the potential influence of CMB. Inspection of the correlation matrix, depicted in Table 1, indicates that the pattern of intercorrelations and their magnitude was as expected, and is consistent with previous studies. The correlation matrix also contains several intercorrelations that are low or even zero. The presence of a strong CMB factor that generally inflates the relationship between constructs seems unlikely.

## Conclusion

To the best of our knowledge, this is the most comprehensive study undertaken to date on the relationship between FFM traits and NFR. The study shows that personality traits (especially ES, but also E and O) should be recognized as important factors in the development of NFR. The magnitude and pattern of both the direct and indirect effects accentuate the complexity of the interaction between personality and other factors in the work environment. Thus, further studies incorporating personality and other work-related factors in the understanding of NFR are needed.

## Supplementary Information

ESM 1 (DOCX 21 kb)

## Data Availability

Available upon request.
