# Supplementary material for: Put your feet up: The impact of personality traits, job pressure, and social support on the need for recovery after work
Source: Curr Psychol. 2022 Mar 14:1–17. Online ahead of print. doi: 10.1007/s12144-022-02950-1 (PMC8918590; doi:10.1007/s12144-022-02950-1)
Supplement: Supplementary file 1 — (DOCX 21 kb) [file 12144_2022_2950_MOESM1_ESM.docx]

Supplemental table 1.

Goodness-of-fit statistics for confirmatory factor analyses (CFA) of measurement models of latent variables included in the study.

| Latent variable | χ2 | *df* | CFI | TLI | RMSEA | [90% CI] | SRMR |
| --- | --- | --- | --- | --- | --- | --- | --- |
| Extraversion | 223.297** | 20 | .89 | .85 | .122 | [.108, .137] | .063 |
| Agreeableness | 178.520** | 27 | .83 | .78 | .091 | [.078, .104] | .061 |
| Conscientiousness | 126.282** | 27 | .93 | .91 | .077 | [.065, .090] | .046 |
| Emotional stability | 170.334** | 20 | .91 | .88 | .105 | [.091, .120] | .051 |
| Openness to experience | 448.643** | 35 | .80 | .75 | .132 | [.121, .143] | .089 |
| Perceived job pressure | 250.209** | 35 | .89 | .85 | .095 | [.084, .106] | .055 |
| Perceived social support | 508.856** | 35 | .84 | .80 | .141 | [.130, .152] | .068 |
| Need for recovery after work | 255.283** | 44 | .89 | .87 | .084 | [.074, .094] | .052 |

**p* < .05, ***p* < .01

Supplemental table 2.

Goodness-of-fit statistics for confirmatory factor analyses (CFA) of full measurement models of the five-factor model (FFM).

| Measurement model | χ2 | df | CFI | TLI | RMSEA | [90% CI] | SRMR |
| --- | --- | --- | --- | --- | --- | --- | --- |
| FFM uncorrelated factors | 4351.207** | 902 | .67 | .66 | .075 | [.073, .077] | .137 |
| FFM correlated factors | 3907.672** | 892 | .71 | .70 | .071 | [.068, .073] | .092 |
| FFM 1-factor | 7784.048** | 902 | .35 | .32 | .106 | [.104, .108] | .128 |

**p* < .05, ***p* < .01

Supplemental table 3.

Goodness-of-fit statistics for confirmatory factor analyses (CFA) of full measurement models.

| Measurement model | χ2 | *df* | CFI | TLI | RMSEA | [90% CI] | SRMR |
| --- | --- | --- | --- | --- | --- | --- | --- |
| Full uncorrelated model | 8242.845** | 2700 | .70 | .69 | .055 | [.054, .056] | .114 |
| Full correlated model | 7458.499** | 2672 | .73 | .74 | .051 | [.050, .053] | .071 |
| Full 1-factor model | 16510.988** | 2700 | .26 | .24 | .087 | [.085, .088] | .116 |

**p* < .05, ***p* < .01
